# Extrusion Improves the Antihypertensive Potential of a Kabuli Chickpea (*Cicer arietinum* L.) Protein Hydrolysate

**DOI:** 10.3390/foods11172562

**Published:** 2022-08-24

**Authors:** Jeanett Chávez-Ontiveros, Cuauhtémoc Reyes-Moreno, Giovanni Isaí Ramírez-Torres, Oscar Gerardo Figueroa-Salcido, Jesús Gilberto Arámburo-Gálvez, Alvaro Montoya-Rodríguez, Noé Ontiveros, Edith Oliva Cuevas-Rodríguez

**Affiliations:** 1 Integral Postgraduate Program in Biotechnology, Faculty of Chemical and Biological Sciences, Autonomous University of Sinaloa, Ciudad Universitaria, Culiacan 80010, Mexico; 2 Postgraduate Program in Food Science and Technology, Faculty of Chemical and Biological Sciences, Autonomous University of Sinaloa, Ciudad Universitaria, Culiacan 80010, Mexico; 3 Faculty of Physical Education and Sports, Autonomous University of Sinaloa, Culiacan 80013, Mexico; 4 Nutrition Sciences Postgraduate Program, Faculty of Nutrition Sciences, Autonomous University of Sinaloa, Culiacan 80019, Mexico; 5 Postgraduate Program in Health Sciences, Division of Biological and Health Sciences, University of Sonora, Hermosillo 83000, Mexico; 6 Clinical and Research Laboratory (LACIUS, URS), Department of Chemical, Biological, and Agricultural Sciences (DC-QB), Division of Sciences and Engineering, University of Sonora, Navojoa 85880, Mexico

**Keywords:** chickpea, hydrolysate, extrusion, antihypertensive, ACE-I, Kabuli, hypertension

## Abstract

Chickpea hydrolysates could have antihypertensive potential, but there are no evaluations in vivo. Thus, the antihypertensive potential of a chickpea protein hydrolysate obtained before and after extrusion (a process that modifies protein digestibility) was evaluated. Protein precipitates were obtained from extruded and unextruded chickpea flours by isoelectric precipitation and hydrolyzed (α-amylase/pepsin/pancreatin). Chemical composition was determined (standard methods). ACE-I inhibition assays were carried out using a colorimetric test. For antihypertensive effect evaluations, spontaneously hypertensive rats (*n* = 8) received the treatments intragastrically (extruded or unextruded hydrolysate (1.2 g/kg), captopril (25 mg/kg), or water only). Fat, ash, and carbohydrate contents were lower in extruded chickpea flour (*p* < 0.05 versus unextruded). The protein content varied between protein precipitates (91.03%/78.66% unextruded/extruded (dry basis)) (*p* < 0.05). The hydrolysates’ IC50 values (mg/mL) were 0.2834 (unextruded)/0.3218 (extruded) (*p* > 0.05). All treatments lowered the blood pressure (*p* < 0.05 vs. water). The extruded hydrolysate showed a more potent antihypertensive effect than the unextruded one (*p* < 0.05), an effect similar to captopril (*p* > 0.05). The results suggest that protein extrusion can be used to generate protein hydrolysates with improved health benefits. The findings have implications for the design and production of functional foods that could help to prevent hypertension or serve as an adjunct in its treatment.

## 1. Introduction

More than 1.2 billion adults have hypertension, and millions die every year due to diseases associated with high blood pressure such as ischemic heart disease, hemorrhagic or ischemic stroke, chronic kidney disease, and other cardiovascular diseases [1]. Common drugs to treat hypertension include lisinopril, captopril, and enalapril, which exert their effect by inhibiting the catalysis of the angiotensin I-converting enzyme (ACE-I). This inhibition prevents the production of the vasoconstrictor angiotensin II from angiotensin I. Although ACE-I inhibitor drugs are cost-effective, they can trigger allergy-like symptoms, such as skin rashes and angioedema, in susceptible individuals [2]. Alternatively, food-derived peptides or hydrolysates have shown the potential to inhibit ACE-I in vitro and to lower blood pressure in spontaneously hypertensive rats [3,4,5]. Therefore, there is a growing interest in testing in vivo protein hydrolysates from different sources obtained after digestion with different enzymes in the search for effective and side-effect-free blood pressure-lowering natural compounds [6,7].

Chickpea is a legume or pulse that contains around 20–22% of highly bioavailable protein with a balanced amino acid profile [8]. In silico and in vitro studies have highlighted that chickpea hydrolysates or peptides obtained with gastrointestinal or non-gastrointestinal enzymes—such as pepsin, trypsin, alcalase, and flavourzyme—can inhibit ACE-I [9,10,11,12], but their antihypertensive potentials have not been evaluated yet. Notably, globulins account for 60–80% of the extractable protein fraction from chickpeas, but the globular protein structure might be a limiting factor for in vitro proteolysis or even digestion in mammals [13]. Heat processing—such as cooking, radiation, roasting, and extrusion—can enhance protein digestibility, increasing the bioavailability and bioactivity of proteins and peptides [14]. Extrusion is a versatile and low-cost process that uses high temperatures for a short time to generate fully cooked food products [15], and it could impact the functionality and bioavailability of proteins as well as the peptide profile obtained from the food matrix [7,14,16]. Thus, the aim of the present study was to evaluate for the first time in vivo the antihypertensive effect of a chickpea protein hydrolysate obtained before and after protein extrusion.

## 2. Materials and Methods

### 2.1. Chickpea Seeds

**Figure 1:**
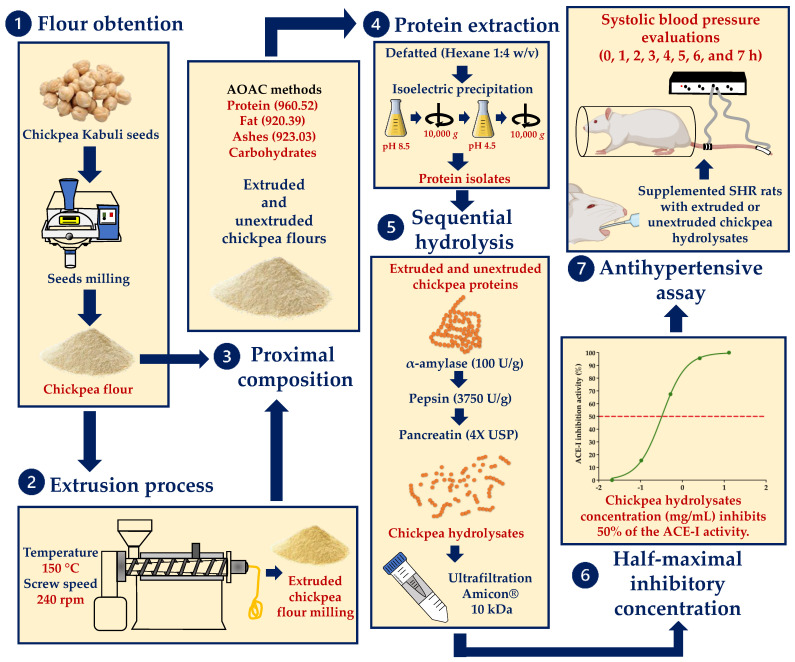
Workflow employed to evaluate the ACE-1 inhibition and antihypertensive effects in vivo of extruded and unextruded chickpea protein hydrolysates.

Chickpea genotype ICC 3421 (Kabuli type, beige) was grown at the Experimental Station of the National Research Institute for Forestry, Agriculture, and Livestock (INIFAP), in Culiacan, Sinaloa, Mexico. Chickpea seeds were harvested in April 2018, cleaned, and stored at 4 °C. Figure 1 summarizes all the assays accomplish to evaluate the ACE-I inhibition and antihypertensive effects in vivo of extruded and unextruded chickpea protein hydrolysates.

### 2.2. Chickpea Flour Preparation

Flours were obtained from whole chickpea seeds using a Model 4 Wiley^®^ Laboratory Mill (Thomas Scientific, Swedesboro, NJ, USA). Individual samples of flour (500 g) were packed in polyethylene bags and stored at 4 °C until analysis.

### 2.3. Extrusion Process

Two hundred and fifty grams of chickpea flour was conditioned (moisture content of 28% (*w*/*w*)), packed in polyethylene bags, and stored at 4 °C for 12 h. The chickpea flours were tempered at 25 °C for 4 h before extrusion. Extrudates were prepared using a single-screw laboratory extruder Model 20 DN (CW Brabender Instruments, Incorporation, South Hackensack, NJ, USA) with a 19 mm screw diameter, a length-to-diameter ratio of 20:1, a nominal compression ratio of 2:1, and a 3 mm die opening. The inner barrel was grooved to ensure zero slip at the wall. The feed rate was 30 rpm. Extrusion temperature of 150 °C and screw speed of 240 rpm were previously optimized processing conditions. The extrudates were cooled (25 °C) and milled using a Lab Mill 3100 (Perten Instruments Mill 3100 Sample Grinder, Champaign, IL, USA), to pass through a 100-US mesh (0.150 mm) screen, packed in plastic bags, and stored at 4 °C [17].

### 2.4. Extruded and Unextruded Chickpea Flours Proximate Composition

Proximate composition was evaluated following the official methods of the Association of Official Analytical Chemist (AOAC) [18]. Moisture was determined according to method 925.10. Crude protein content was evaluated using the micro-Kjeldahl assay (method 960.52). Fat was determined according to the method 920.39 using a Soxtec System HT 1043 Extraction Unit (Soxhlet apparatus) and petroleum ether as solvent. Incineration at 550 °C was used to determine ashes (method 923.03). Carbohydrates were estimated by difference of the other components. All determinations were performed in triplicate and the results were expressed in g/100 g of dry weight.

### 2.5. Extraction and Concentration of Chickpea Protein

Extruded and unextruded chickpea flours were defatted with hexane (1:4 *w*/*v*; constant stirring at 500 rpm for 4 h) and the defatted samples dried overnight at 25 °C. To extract protein, 200 g of defatted flour were resuspended in distilled water (1:10, *w*/*v*), the pH adjusted to 8.5 (NaOH, 1 M), and the mixture stirred at 500 rpm for 2 h. The flour suspensions were centrifuged at 10,000× *g*/10 min. Afterwards, the supernatants were collected, and the pellets were washed again under the same conditions. The pH of the supernatants was adjusted to 4.5 (HCl, 1 M) and the solutions stirred at 500 rpm for 2 h to promote protein precipitation. Finally, the protein suspensions were centrifuged at 10,000× *g*/10 min and the pellets lyophilized (protein isolates/concentrates) and stored at −20 °C until their use [19,20]. Protein concentration was estimated following the AOAC method 960.52.

### 2.6. Hydrolysis and Fractioning of Chickpea Protein

Extruded and unextruded chickpea protein isolates/concentrates were hydrolyzed as previously described with minor modifications [20]. Briefly, in a final volume of 100 mL of a 0.005 M phosphate (NaKH_2_PO_4_ and Na_2_HPO_4_) buffer solution at pH 6.9, 3 g of the protein isolates/concentrates were treated with α-amylase (100 U/g) from porcine pancreas (EC 3.2.1.1., ≥10 units/mg solid). The enzymatic reactions were carried out at 37.5 °C for 5 min with stirring at 200 rpm. Afterwards, the pH of the solutions was adjusted to 2 and pepsin (3750 U/g) from porcine gastric mucosa (EC 3.4.23.1, ≥250 units/mg solid) was added. Pepsin digestion was carried out for 60 min at 37 °C at 200 rpm. Then, the pH of the solutions was adjusted to 6.5 and pancreatin (10 mg/g; 4X USP) from porcine pancreas was added. Pancreatin digestions were carried out for 120 min at 37 °C at 200 rpm. The hydrolysates were centrifuged at 20,000× *g* for 10 min at 4 °C, and the supernatants were collected. The samples were fractionated by ultrafiltration using Amicon^®^ tubes with a cut-off of 10 kDa (Millipore, MA, USA). The permeates were collected, lyophilized, and stored at −20 °C for further analysis.

### 2.7. Half-Maximal Inhibitory Concentration

The angiotensin converting enzyme (ACE-I) half-maximal inhibitory concentration (IC 50) was determined using the ACE Kit-WST kit (Dojindo Molecular Technologies, Inc., Kumamoto, Japan) following the manufacturer’s instructions. The protein concentration of chickpea hydrolysates was determined using the DC protein assay (Bio-Rad Laboratories, Hercules, CA, USA) for microplates. A bovine serum albumin standard curve was constructed for protein quantification. IC 50 was defined as the chickpea hydrolysate concentration (mg/mL) required to cause 50% of the ACE-I inhibition.

### 2.8. Animals and Ethical Aspects

Antihypertensive assays were carried out using male spontaneously hypertensive rats (SHRs) aged 12/13 weeks old and weighing 250–300 g body weight. The SHRs were acquired from the National Autonomous University of Mexico (UNAM, Cell Physiology Institute). The rats were kept in plastic cages with stainless steel wire lids at 24 °C with 12 h light/dark cycles. The animals were fed with a commercial diet (Rodent Lab Chow 5001). Food and water were available ad libitum. The experimental protocols were approved by the Ethics Review Board of the Autonomous University of Sinaloa (CE-UACNYG-2015-SEP-001).

### 2.9. Effect of Chickpea Hydrolysates on Blood Pressure in SHRs

Eight SHRs were supplemented with chickpea hydrolysate (1.2 g/kg body weight), either extruded or unextruded. As positive and negative controls, captopril solubilized in water (25 mg/kg body weight) and water alone were administered, respectively [4]. All treatments were administered intragastrically to each animal by using sterile plastic feeding tubes (18GA × 75 mm, Instech Laboratories, Inc., Plymouth Meeting, PA, USA). Wash out periods of 48 h were implemented after the administration of treatments. The SBP evaluations were carried out before (time 0) and after treatments (1, 2, 3, 4, 5, 6, and 7 h) by using a CODA tail-cuff blood pressure monitor (Kent Scientific Corp., Torrington, CT, USA).

### 2.10. Statistical Analysis

Statistical analyses were carried out using the statistical software GraphPad Prism 8.0. (GraphPad Software, San Diego, CA, USA). Data distribution was determined using the Shapiro–Wilk test. Differences in proximate composition and soluble protein were assessed using unpaired *t*-tests. IC50 values were compared by the extra-sum-of-squares F test of non-linear regression lines. Differences in systolic blood pressure among treatments were assessed using the Friedman test followed by the two-stage linear step-up procedure of Benjamini, Krieger, and Yekutieli for multiple comparisons. Statistical significance was considered as a *p*-value < 0.05.

## 3. Results and Discussion

### 3.1. Protein Extraction and Proximate Composition

Table 1 shows the results on a dry basis of the proximate analysis of unextruded and extruded chickpea flours. On one hand, extruded (23.92%) and unextruded (24.04%) chickpea flours had quite similar protein content (*p* > 0.05), which means that the extrusion process has no impact on the content of this component. On the other hand, the protein content of the chickpea flours was slightly lower [7,21] or higher [22,23,24] than the mean values reported for this legume (from 18.5% to 25.0%). For the present study, chickpea seeds were obtained from plants that were grown in optimal conditions in an experimental station, and the environment provided could influence the protein content of the seeds. Furthermore, it should be considered that the protein content of chickpea seeds varies among cultivars [25] and that the methodological approaches utilized for protein quantification could vary among studies. Regarding fat and ash content, these components were higher in the extruded flour than in the unextruded one (*p* < 0.05). Others have reported opposite results, which depend on the extrusion conditions [16]. In contrast to the fat and ash content, the extrusion process decreased the carbohydrate content (*p* < 0.05) (Table 1). In general, the extrusion process promotes several changes in the proximate composition of food matrices. The changes—such as less water activity, the formation of carbohydrate–lipid and protein–lipid complexes and resistant starch, and reduction in tannins and phytates—have been related to the operation conditions of the extruder (temperature, screw speed, and high shear force) [26,27].

The precipitates obtained by using the isoelectric precipitation method had protein contents of 91.03% and 78.66% (*w*/*w*) (*p* < 0.05), for the ones obtained from unextruded and extruded defatted chickpea flours, respectively. Others have reported protein contents of 78.53%, 69.84%, and 83.31% in unextruded chickpea concentrates [7,10,24]. As stated before, the differences in protein content could be attributed to the genetic background of the cultivars used [28] and/or changes in the methodologies applied for the quantification of the food components.

### 3.2. Determination of the IC50

**Figure 2:**
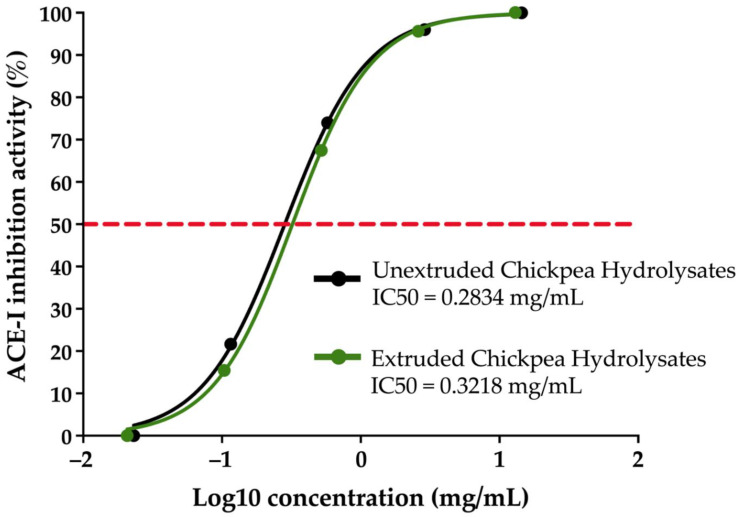
IC50 determination of chickpea hydrolysates. Extra-sum-of-squares F test of non-linear regression lines of IC50 estimations was employed to compare the two IC50 values obtained.

Extruded and unextruded chickpea proteins were submitted to enzymatic hydrolysis using sequential digestion with α-amylase, pepsin, and pancreatin to obtain chickpea hydrolysates with ACE-I inhibitory activity. The kinetics for obtaining the IC50 values of the chickpea hydrolysates are shown in Figure 2. Extruded and unextruded chickpea hydrolysates showed IC50 values of 0.3218 and 0.2834 mg/mL, respectively (*p* > 0.05). At a concentration of 12.97 and 14.45 mg/mL, the extruded and unextruded chickpea hydrolysates showed the highest ACE-I inhibition (97.40% and 98.24%, respectively). Others reported lower IC50 values of chickpea protein hydrolysates of the Kabuli variety (0.229 mg/mL) than the ones found in the present study, but the sequential digestion of the protein isolate/concentrate was carried out using pepsin, trypsin, and α-chymotrypsin [12]. The IC50 values reported using non-digestive enzymes were 0.282 mg/mL (papain) and 0.316 mg/mL (alcalase/flavourzyme), which are similar to the IC50 values determined in the present study (0.3218 and 0.2834 mg/mL, for extruded and unextruded chickpea hydrolysates, respectively). By using the sequential digestion alcalase/flavourzyme, an IC50 value of 0.19 mg/mL of a chickpea protein hydrolysate was reported, but the chickpea seeds were of the cultivar Athenas [29]. IC50 values of 0.000669 and 0.000030 mg/mL have been reported for protein hydrolysates of unextruded chickpea (cv. Kabuli) protein concentrates obtained after digestions with alcalase or papain, respectively [10]. These are the lowest ACE-I inhibition IC50s ever reported for chickpea protein hydrolysates. Our results (IC50 values of 0.3218 mg/mL (extruded) and 0.2834 mg/mL (unextruded)) are at least 423-fold higher than the IC50 values previously mentioned [10], which are only 2–16.7-fold higher than the IC50 values reported for captopril using synthetic substrates [30], but they are not so far from the IC50 values of protein hydrolysates of different sources that have shown antihypertensive potential in murine models [3,4,31]. In general, our in vitro evaluations of ACE-I inhibition suggest that the extrusion process negatively or not impacts the potential ACE-I-associated antihypertensive properties of chickpea protein hydrolysates obtained after the sequential digestion of the proteins with α-amylase, pepsin, and pancreatin. However, others have reported that the extrusion process can improve the ACE-I inhibitory potential of protein hydrolysates [32]. Thus, in vivo studies using extruded-protein hydrolysates are required to solve this dichotomy or provide insights into a potential matrix-dependent ACE-I-inhibitory effect.

### 3.3. Effect of Unextruded and Extruded Chickpea Protein Hydrolysates on Blood Pressure

**Figure 3:**
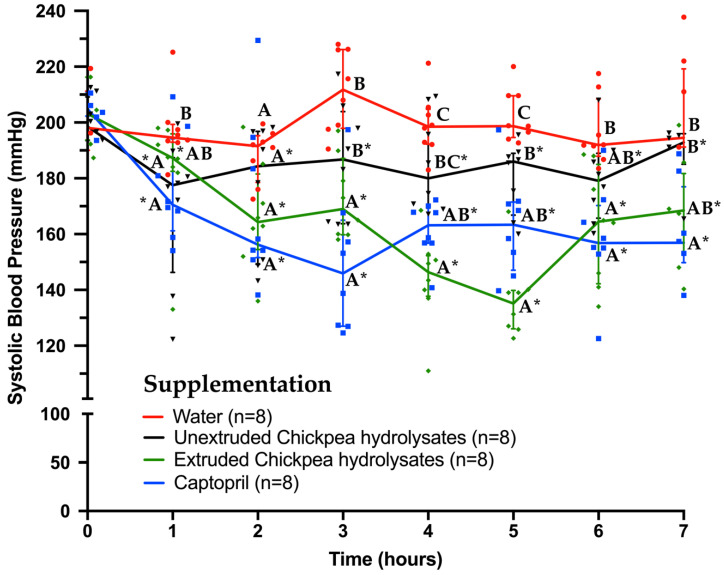
Systolic blood pressure in spontaneously hypertensive rats before (time zero) and after (times 1 to 7 h) supplementation with water (1.0 mL), captopril (25 mg/kg), extruded, and unextruded chickpea hydrolysates (1.2 g/kg each). Horizontally, asterisks in each time point indicate statistically significant differences from their respective basal values (time zero) (*p* < 0.05). Vertically, different letters at each time point mean statistically significant differences between groups (*p* < 0.05). Red dots (water group), black triangles (unextruded Chickpea hydrolysate group), green rhombus (extruded Chickpea hydrolysate group), and blue squares (captopril group) represent individual systolic blood pressure values from each rat. Data are presented as medians and interquartile ranges.

There is compelling evidence about the antihypertensive potential of food-derived peptides from different food matrices. About 20 years ago, it was reported that chickpea hydrolysates or peptides can inhibit ACE-I in vitro [11,29], but the antihypertensive potential of such hydrolysates or peptides remains uncertain. The impact of heat processing of chickpea proteins on the generation of hydrolysates with the potential to inhibit ACE-I in vitro and lower blood pressure has not been evaluated either. The present study provides information about the antihypertensive potential of a chickpea-protein hydrolysate obtained before (unextruded) and after cooking the protein matrix by an extrusion process. Spontaneously hypertensive rats were administered intragastrically an unextruded or extruded chickpea hydrolysate; and, as negative and positive controls, water or captopril, respectively. The hypertensive rats lowered their systolic blood pressure after the first hour of the administration of either unextruded or extruded chickpea hydrolysates (Figure 2). These antihypertensive effects were between −10.92 and −20.45 mmHg (5.5–10.45% reduction) and between −9.16 and −61.41 mmHg (4.57–31.42% reduction), for the unextruded and extruded hydrolysates, respectively (*p* < 0.05 for both hydrolysates compared to basal values (time zero)) (Figure 3). The antihypertensive effects of the hydrolysates were sustained at all time points throughout the experiments, in line with the effect induced by the administration of captopril (*p* < 0.05 for all treatments, except water, compared to basal values (time zero)) (Figure 3). Similar antihypertensive effects have been reported using food-protein hydrolysates derived from sources others than chickpeas, such as amaranth (about 59 mmHg of systolic blood pressure reduction after 6 h of the hydrolysate administration; [4], flaxseed (−29 mmHg at 4 h [33]), lima bean (51% systolic blood pressure reduction after two weeks of daily supplementation of the hydrolysates; [34]), velvet bean (8.84–27.29% of systolic blood pressure reduction after 0–140 min of the hydrolysates administration; [35]), rice bran (−34 mmHg systolic blood pressure reduction after 6 h of the hydrolysate administration; [36]), and pigeon pea (−33 mmHg after 4 h of the hydrolysate administration [37]). Overall, the results show that both the unextruded and the extruded chickpea protein hydrolysates not only have the potential to inhibit ACE-I in vitro, but also have the potential to lower the systolic blood pressure in spontaneously hypertensive rats. Furthermore, the antihypertensive effects can last at least seven hours, which supports the notion that the antihypertensive chickpea peptides generated are not only bioavailable but also have a half-life in circulation long enough to sustain an antihypertensive effect for a significant period.

Contrary to the ACE-I inhibition in vitro assays, the magnitude of the antihypertensive effect of each treatment showed that the extruded chickpea protein concentrate can generate a hydrolysate with improved antihypertensive potential. In fact, the extruded chickpea hydrolysate showed antihypertensive effects similar to the ones triggered by captopril at all times after the treatments administration (*p* > 0.05) (Figure 3). Furthermore, the extruded hydrolysate showed a more potent antihypertensive effect than the unextruded one, especially between 3 and 5 h after their administration (−29.9, −61.4, and −61.9 vs. −15.9, −19.5, and −20.0 mmHg, respectively) (*p* < 0.05) (Figure 3). Certainly, the extrusion-associated physicochemical changes at the protein level include not only a decreased protein dispersibility index and an increase in beta-sheet structure, which indicates reduced solubility and potential protein aggregation, but also an increase in protein digestibility [16]. In this context, extrusion makes proteins more accessible to peptide bond cleavage by gastrointestinal enzymes [16,38,39], which in turn could improve the protein quality parameter protein digestibility-corrected amino acid score as the content of limiting amino acids in chickpea protein hardly differs before and after extrusion [21]. Furthermore, protein denaturation and aggregation make them more susceptible to enzymatic hydrolysis due to the target sites becoming more exposed [40]. This implies that different patterns of antihypertensive peptides could be generated during the proteolytic digestion of extruded and unextruded proteins [38] and this can impact the antihypertensive potential of the hydrolysates generated.

In the present study, the hydrolysates utilized for in vitro and in vivo assays underwent amylolytic digestion and were filtered through a 10 kDa cut-off membrane. Amylolytic digestion can help to solubilize proteins that are buried (starch granule-channel proteins) or on the surface of starch granules (starch granule-surface proteins) [41], making them more accessible for proteolytic enzymes. Consequently, this treatment is expected to increase soluble protein content and increase the efficiency of proteolytic catalysis. In this context, peptides with molecular masses of less than 10 kDa are expected to be generated since extruded and unextruded flour hydrolysates obtained after digestion with pepsin and pancreatin generate peptides with molecular masses between 0.5 and 4.1 kDa [38]. Furthermore, passing the hydrolysates through a 10 kDa membrane, the amylolytic and proteolytic enzymes are removed, leaving most—or all—of the peptides with the potential to inhibit ACE-I [42]. Notably, this process does not generate significant changes in soluble protein content between extruded and unextruded protein hydrolysates (69.74% vs. 71.88%, respectively *p* > 0.05). The potential mechanisms that underlie the hydrolysate/peptide-induced hypotensive effects could be related to different targets. For instance, a couple of axes exert opposite effects in the renin–angiotensin–aldosterone system, and they are associated with different key molecules—such as ACE-I, angiotensin and angiotensin receptor type 1, ACE-II, angiotensin-(1-7), and Mas-receptor, among others—to regulate vasoconstriction or vasorelaxant activity [43]. The present study demonstrates that chickpea protein hydrolysates can inhibit ACE-I in vitro and that the hydrolysates can lower the systolic blood pressure in spontaneously hypertensive rats. However, it remains uncertain if ACE-I inhibition is the only or the main mechanism by which the hydrolysates can exert a hypotensive effect in an animal model of hypertension, or if some extruded or unextruded chickpea peptides can inhibit protein–protein interactions and regulate blood pressure. As stated by others [44], studies addressing the changes in gene expression after chronic hydrolysate consumption can help to deepen our understanding of this topic.

## 4. Conclusions

This is the first study to evaluate the antihypertensive potential of a chickpea protein hydrolysate. The hydrolysate can efficiently lower the systolic blood pressure in spontaneously hypertensive rats. Notably, the extrusion of chickpea proteins increased the antihypertensive potential of the hydrolysate, suggesting that extrusion leads to a different profile of antihypertensive peptides. The findings have implications for producing ingredients for functional foods that could help to prevent hypertension or serve as an adjunct in the treatment of this non-communicable disease. Further studies directed to identifying the peptide profile of extruded and unextruded chickpea protein hydrolysates and their analysis under an in silico approach proposed previously (simulated enzymatic hydrolysis and ADMET prediction followed by molecular docking [9]) are warranted.

## Figures and Tables

**Table 1:** Proximate composition of unextruded and extruded chickpea flours (dry basis).

Chickpea Flour	Protein (g/100 g)	Fat (g/100 g)	Ash (g/100 g)	Carbohydrates (g/100 g)
Unextruded	24.04 ± 0.25 ^a^	3.60 ± 0.11 ^a^	3.36 ± 0.03 ^a^	69.00 ± 0.17 ^a^
Extruded	23.92 ± 0.18 ^a^	4.73 ± 0.49 ^b^	3.63 ± 0.03 ^b^	67.72 ± 0.68 ^b^

Values in columns with different literals show statistically different values (*p* < 0.05). Mean values ± standard deviations are shown.

## Data Availability

Data are available within the article.
